# The potential role of purinergic signaling in cancer therapy: perspectives on anti-CD73 strategies for prostate cancer

**DOI:** 10.3389/fimmu.2024.1455469

**Published:** 2024-09-17

**Authors:** Carla Fernanda Furtado Gardani, Fernando Mendonça Diz, Luísa Brandalise Dondé, Liliana Rockenbach, Stefan Laufer, Fernanda Bueno Morrone

**Affiliations:** ^1^ Escola de Medicina, Programa de Pós-Graduação em Medicina e Ciências da Saúde, Pontifícia Universidade Católica do Rio Grande do Sul, Porto Alegre, Brazil; ^2^ Laboratório de Farmacologia Aplicada, Escola de Ciências da Saúde e da Vida, Pontifícia Universidade Católica do Rio Grande do Sul, Porto Alegre, Brazil; ^3^ Centro de Pesquisa Pré-Clínica, Instituto do Cerebro do Rio Grande do Sul (InsCer), Pontíficia Universidade Católica do Rio Grande do Sul, Porto Alegre, Brazil; ^4^ Department of Pharmaceutical and Medicinal Chemistry, Institute of Pharmacy, Eberhard Karls University of Tübingen, Tübingen, Germany; ^5^ Escola de Ciências da Saúde e da Vida, Programa de Pós-Graduação em Biologia Celular e Molecular, Pontifícia Universidade Católica do Rio Grande do Sul, Porto Alegre, Brazil

**Keywords:** adenosine, CD73, CD39, ectonucleotidases, purinergic system, prostate cancer

## Abstract

Purines and pyrimidines are signaling molecules in the tumor microenvironment that affect cancer immunity. The purinergic signaling pathways have been shown to play an important role in the development and progression of cancer. CD39 and CD73 are ectonucleotidases responsible for breaking down ATP or ADP into adenosine, which regulates immunosuppression in various types of cancer. These enzymes have been studied as a potential therapeutic target in immunotherapy, and recent research suggests a correlation between ectonucleotidases and clinical outcomes in cancer.Prostate cancer is the most diagnosed cancer in men, after non-melanoma skin tumors, and is the second leading cause of death in men in the world. Despite having long survival periods, patients often receive excessive or insufficient treatment. Within this complex landscape, the adenosine/CD73 pathway plays a crucial role. Therefore, this review aims to highlight new findings on the potential role of purinergic signaling in cancer treatment and emphasizes the importance of anti-CD73 as a pharmacological strategy for prostate cancer therapy.

## 1 Introduction

Prostate cancer (PC) is the most frequently diagnosed urological neoplasm in males and it has high incidence today, being the second most common type of cancer and one of the leading causes of cancer-related death (1). Among the risk factors for the development of PC: are advanced age, family history, and genetic mutations such as BRCA1/2 mutations. According to a study published by the World Cancer Research Fund International, certain lifestyles such as excessive exposure to tobacco, obesity, a high-fat diet, and a sedentary lifestyle also contribute to unfavorable outcomes (2).

PC treatment is a challenge for biomedical sciences and has a great impact on public health, as it presents a high incidence and prevalence today (3, 4). Despite having long survival periods, patients often receive excessive or insufficient treatment due to inadequate risk stratification (5, 6).

The purinergic signaling pathways have been shown to play an important role in the development and progression of cancer (7). Purines and pyrimidines are signaling molecules in the tumor microenvironment that affect cancer immunity (8). Ectonucleotidases, such as CD39 and CD73, hydrolyze ATP or ADP into AMP and adenosine, respectively, regulating the nucleosides’ impact on immunosuppression in various tumor types (7). Besides, CD73 and CD39 are overexpression of several cancers (7, 9). Studies have suggested that the hydrolysis of nucleotides, which produce adenosine in the bloodstream, may be associated with prostate cancer progression and promote cancer cell growth (10). Targeting the ATP-adenosine pathway and CD39 reduces immunosuppressive adenosine accumulation and increases pro-inflammatory ATP levels (11).

Additionally, the role of CD73 has been investigated as a potential target for cancer immunotherapy (12). Monoclonal antibodies and small molecule inhibitors that interfere with the adenosine/CD73 pathway are being investigated in clinical trials (13). According to a previous study, it was demonstrated that there is a difference in the nucleotide hydrolysis profiles between patients with PC and healthy controls (10). However, further research is required to determine the association between purinergic signaling modulation and cancer. Understanding the factors that influence tumor progression and the correlation between CD39 and CD73 expression and clinical outcomes of prostate cancer is crucial. Therefore, establishing new strategies to improve the overall survival of PC patients is essential. In this review, we specifically focus on purinergic signaling, particularly anti-CD73, as a treatment for prostate cancer.

## 2 The involvement of purinergic signaling pathways in cancer development

Purinergic signaling was initially proposed as a complex network that could regulate various cellular processes, such as proliferation, differentiation, and cell death (14). Later, the purinergic receptors were identified and classified into two groups: adenosine or P1 receptors and ATP or P2 receptors (15). The P1 receptors are G protein-coupled and can be further divided into four types (A_1_, A_2A_, A_2B_, and A_3_) based on their molecular structure, biochemistry, and pharmacology (14, 15). On the other hand, P2 receptors can be divided into P2Y receptors, which are activated by ATP and ADP and are also G protein-coupled. There are eight subunits of P2Y receptors (1, 2, 4, 6, 11, 12, 13, and 14) (14, 16). Additionally, there are P2X receptors, which are ATP-responsive ion channels that consist of trimeric forms of different subunits. The P2X subunits are classified from P2X1 to P2X7 according to their historical order of cloning, and each subunit has unique pharmacological and physiological properties (17, 18).

The ectonucleotidases play a crucial role in regulating the effects of extracellular nucleotides. These enzymes form a family that works in a highly coordinated manner to break down nucleotides and convert them into nucleosides, which is essential for extracellular purinergic regulation (15, 19). This family of enzymes includes the following members: Ecto-nucleoside triphosphate di phosphohydrolases (NTPDases –EC 3.6.1.5); Ecto-nucleoside pyrophosphatase/phosphodiesterases (NPPs – EC 3.6.1.9/EC 3.1.4.1); Alkaline Phosphatase (AP – EC 3.1.3.1); Ecto-5’-nucleotidase/CD73 (ecto-5’NT/CD73 - Ec 3.1.3.5). E-NTPDases hydrolyze ATP to ADP and ADP to AMP, while ecto-5’-nucleotidase acts on the conversion of AMP to ADO. Alkaline phosphatases hydrolyze monophosphate nucleotides and NPPs act in the extracellular catabolism of dinucleotides (20).

The NTPDase1 (E.C. 3.6.1.5) is also called CD39, while ecto-5’-nucleotidase can also be called CD73 (E.C. 3.1.3.5), as they are markers of B and T lymphocyte maturation. CD39 is a member of the NTPDase group and is situated on the surface of the plasma membrane (PM). It is responsible for the conversion of ATP or ADP into AMP extracellularly, which subsequently binds to purinergic system receptors, activating them. In contrast, CD73, also known as ecto-5’-nucleotidase, extracellularly dephosphorylates AMP to ADO, which has a pro-tumor effect and binds to P1 receptors (14, 15, 19, 20).

Maintaining a balance between ATP and ADO is crucial to prevent uncontrolled tissue destruction and excessive inflammatory response. In stressful situations such as inflammation, neoplasms, and ischemia, there is an accumulation of ATP in the extracellular environment (15, 21). However, in healthy tissues, the accumulation of extracellular nucleotides and nucleosides is insignificant except in the synaptic clefts or muscle interstitium during exercise (22). The accumulated extracellular ATP (eATP) can stimulate P2 purinergic receptors (P2X and P2Y), leading to the activation of immune cell inflammation activity (14). Meanwhile, the accumulated extracellular ADO (eADO) can bind to downstream P1 purinergic receptors, which have immunosuppressive effects in many tumor types, including urological tumors, primarily PC (23).

The tumor microenvironment (TME) is a dynamic environment characterized by biochemical and cellular features that play a crucial role in regulating the metabolism, proliferation, and dissemination of tumor cells (23, 24). The TME may exhibit both pro-inflammatory and pro-tumor characteristics, facilitating neoplastic progression. The biochemical composition of the TME results from interactions between host cells and tumor cells, leading to the release of damage-associated molecular patterns (DAMPs) and activation of innate immunity, including the recruitment of immune cells such as macrophages and dendritic cells through the NOD-like receptor (25). The TME is a complex milieu that includes cytokines, growth factors, cytotoxic molecules, proteases, and DAMPs. This environment significantly impairs the host’s immune system’s ability to combat the tumor (24, 25).

In TME, ATP is released through various mechanisms, such as passive efflux due to cell death, active transport via calcium channels, and microvesicles of plasma membranes (25). The TME is a crucial site for producing and releasing extracellular ATP (eATP) and adenosine (ADO), and this pathway’s modulatory actions have become a focal point for targeted cancer therapies (26, 27). The antineoplastic activity of ATP was first described by Rapaport (1983), who demonstrated that the addition of exogenous ATP caused cells to rest in the S cell cycle phase, and cancer cells were treated with ADO which promoted tumor growth (28).

Besides, neoplastic cells show high expression of CD39 as well as immune cells, particularly NK cells, Treg cells, macrophages, and tumor-specific effector T cells in TME (29). Tumor-associated macrophages co-express CD39 and P2X7 receptors, consequently the activation of these receptors by eATP stimulates specific actions in the inflammasome that lead to the release of cytosolic caspases (30). Including the release of microvesicles from monocytes and macrophages, resulting in the rapid release of interleukin-1β and interleukin-18 (31). An *in vivo* study has shown that eATP release promotes the rapid recruitment of neutrophils through the caspase activation mechanism, in addition to binding to P2Y2 receptors, enhancing this recruitment (32). Elevated CD39 expression promotes its pro-inflammatory actions by stimulating the regulatory function of Th17 cells, causing the exhaustion of CD8+ T cells, stimulating neutrophil chemotaxis, suppressing the function of NK cells, stimulating the presentation of dendritic cells (DC) and inhibiting platelet aggregation (33).

**Figure 1:**
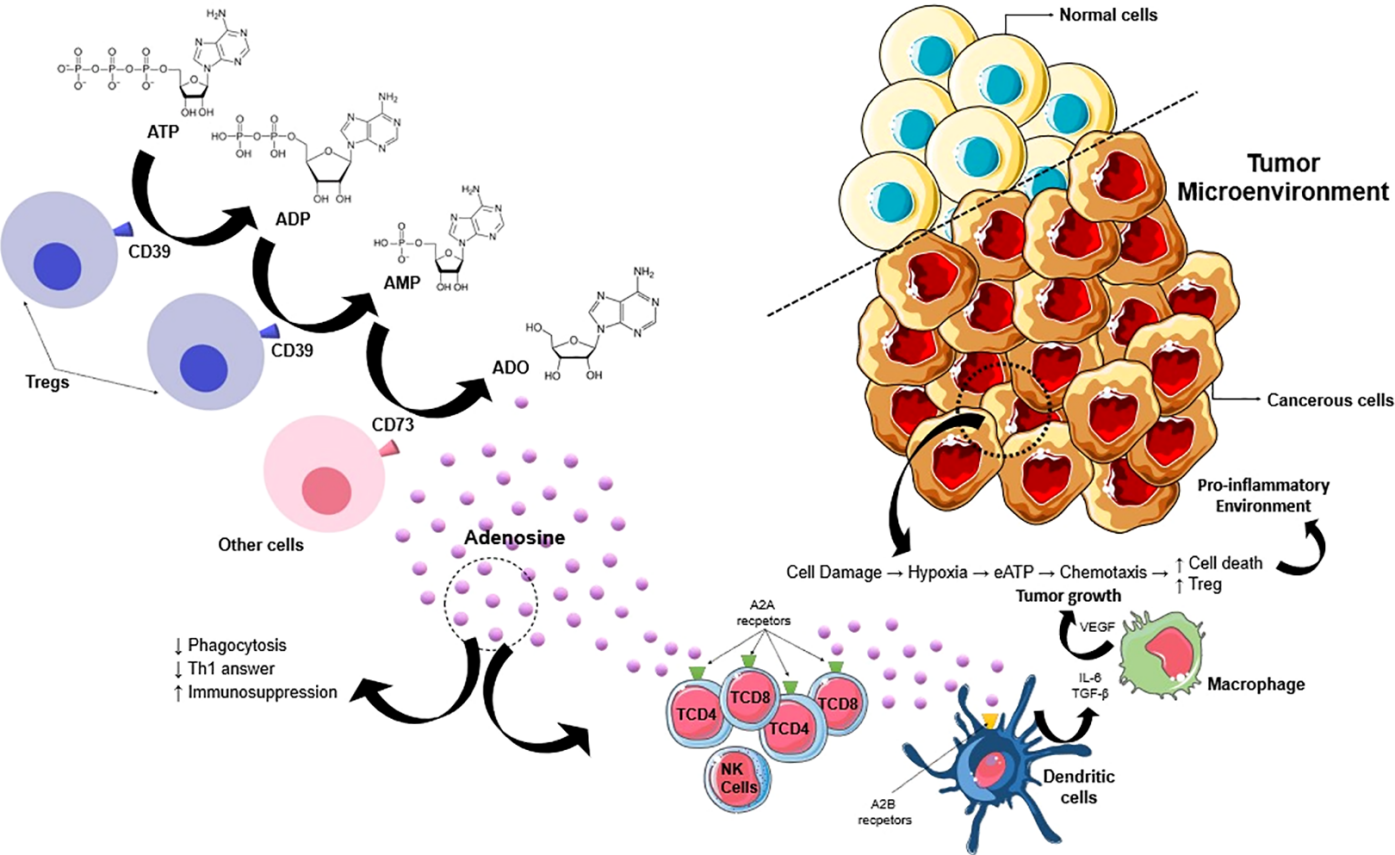
Promising mechanism of purinergic signaling pathway in cancer progression. ATP in TME is released by passive efflux due to cell death, so this ATP is converted into adenosine by upregulated CD39 and CD73. Adenosine promotes cancer progression by acting through P1 receptors (A1, A2A, A2B e A3) and activates the immunosuppressive action of Treg cells. This process enhances the production of pro-angiogenic factors (β-FGF, VEGF, and IL-8). So, the adenosine generated by CD39/CD73 expressed either on primary neoplastic cells or on cancer exosomes generates an immunosuppressive environment by acting on macrophages, neutrophils, dendritic cells, and T cells. IL-8, interleukin-8; VEGF, vascular endothelial growth factor; TGF- β, transforming growth factor beta.

Moreover, targeting CD73 or A_2A_ receptor (A2AR) is necessary for the activation of effector T cells and natural killer (NK) cells in the tumor. Through inhibition of A2BR or CD73, cancer cell survival is significantly hampered (34). Moreover, the understanding of the TME has led to the identification of metabolic pathways essential for tumor cell survival. Tumor hypoxia induces a wide network of metabolic and immunological changes that favor tumor growth and progression, with proangiogenic and immunosuppressive effects. The consequence is the promotion of a strong selection among tumor cells with an increase in their aggressiveness instead of an antitumor response (35). **Figure 1** illustrates the mechanisms that encompass purinergic signaling, particularly the adenosine pathway, and their impact on the tumor microenvironment.

## 3 CD73 expression and activity play a critical role in cancer treatment

CD73 is expressed in different body tissues and on various cell types, such as B lymphocytes, some T lymphocytes, endothelial, epithelial, and tumor cells (34). Tumor cells exhibit several evasion mechanisms to evade the surveillance of the immune system, mainly with the help of inhibitory immune cells and immunosuppressive cytokines. Among immunosuppressive cells, the actions of regulatory T cells (Tregs), macrophages, and natural killer cells (NK) stand out, which together have suppressed antitumor activities and consequent pro-tumor actions (35).

The expression of CD73 by such immune cells creates an immunosuppressive TME, which affects the basic functions of these cells, facilitating the development of tumor cells (36). The Treg cells in humans express low levels of CD73, but it has been documented that their expression may be elevated in cancer patients, consequently, inhibition of CD73 on Treg cells inhibits their immunosuppressive action (37). On the other hand, anergic or exhausted effector T cells exhibit high levels of CD73, consequently, the reduction of this expression in these cells leads to antitumor effects, with potentiation of their effector functions (38). It is already documented that NK cells in TME exhibit high expression of CD73, consequently, the ADO released from AMP dephosphorylation in TME suppresses the functions of NK cells, through the activation of A_2A_ receptors, mainly (39).

Several studies have already shown that CD73 is overexpressed in various types of solid tumors, especially in more aggressive tumors with greater potential for metastasis (40). CD73-deficient mice are resistant to prostate carcinogenesis, suggesting that in the future therapies based on anti-CD73 monoclonal antibodies could dramatically reduce the growth of prostate tumor cells, inhibiting the process of metastasis and unfavorable outcomes (36). Therefore, its high expression in tumor cells is associated with a worse prognosis (36, 37).

However, CD73 can also be under-expressed and will not necessarily lead to a good prognosis. A study showed that when analyzing the expressions of CD73 in immunohistochemical studies of 136 patients with non-metastatic breast cancer and correlating with the clinical evolution, he noticed that patients with CD73 overexpression had a longer disease-free interval and longer global survival (41). Another study also demonstrated that the high expression of CD73 will lead to a better prognosis in patients with epithelial ovarian carcinoma (42). Lung cancer has high expressions of CD73, which promotes tumor growth via the EGFR pathway, consequently, these patients tend to be more resistant to chemotherapy in general (43). Regarding colon cancer, CD73 overexpression correlates with more aggressive behavior/metastasis and poor response to chemotherapy regimens containing 5-fluorouracil (44).

Many factors may contribute to the modulation of CD73 expression in tumor cells (40). Based on this theory, several studies confirmed that the use of APCP (a-b-methyl adenosine-5-diphosphatase), an unhydrolyzed analog of ADP, a selective inhibitor of CD73, inhibits cell proliferation, controlling the advance of the disease (45). Blocking CD73 using APCP has been associated with tumor regression, potentiation of the antitumor T-cell response, and increased survival in animal models (46).

Although blocking with anti-CD73 agents is not curative, inhibition of this pathway improves the antitumor activity of other treatments, such as those related to the CTLA-4 and PD-1 pathways (47). The expectation is that the combination of anti-PD-1 and anti-CD73, or A2A receptor antagonists, will result in more effective immune responses mediated by antitumor T cells (48). Another relevant data regarding CD73 is its solubility, so its activity can be evaluated by its expression in plasma (49). Patients with metastatic melanoma show high basal levels of CD73 expression in their plasma, but after starting treatment with nivolumab, they show a sharp drop, suggesting a possible marker of response to treatment (50).

It appears that anti-CD73 monoclonal antibody (mAb) treatment not only stops tumor growth but also limits the metastasis process through direct inhibition of tumor cell adhesion to endothelial cells. Therefore, the antitumor effects of anti-CD73 mAb or APCP are not limited to blocking CD73 enzymatic activity alone (36). On the other hand, the possible toxicity of anti-CD73 mAbs is still unknown, since there is the expression of CD73 in normal cells in various tissues, so its enzymatic action is also necessary for several physiological processes, which may be a limiting factor to its use (51). Interestingly, Morello et al. (2017) demonstrated that patients with metastatic melanoma who have higher CD73 activities have worse prognoses (50). Besides, researchers found that exosomal CD73 from patients with metastatic melanoma was associated with dampening T-cell activity and potentially influencing the effectiveness of anti-PD-1 immunotherapy (52). Targeting multiple immunosuppressive pathways can synergistically enhance anti-tumor immune responses (29). Inhibition of adenosine signaling has been shown to synergize with anti-PD-1 or anti-CTLA-4 mAbs in preclinical studies (47, 48).

As described in **Table 1**, 34 studies were found, including phases I, II, and III, of which 41% of the studies are only phase I, and 38.3% are phase II, while studies that include phases I and II are present in 17.7%. Only 3% of the studies are only phase III. Among these studies, lung cancer is the most researched clinical study, followed by breast cancer and prostate cancer. In the studies, combinations of different classes are tested together with the main agent, to verify the effectiveness of a combined therapy, the most frequent associations in the studies are, oleclumab, durvalumab, oleclumab + durvalumab and monalizumab (**Table 1**).

**Table 1:** Clinical trials involving CD73 antibodies in cancer treatment.

Drug	Combination	Identification	Phase	Disease	Status
**Durvalumab**	Oleclumab	NCT03773666	I	Bladder cancer	Completed
**Durvalumab**	Oleclumab + Monalizumab	NCT05221840	III	NSCLC	Recruiting
**Paclitaxel + Carboplatin + Durvalumab**	Oleclumab	NCT03616886	I/II	Triple Negative Breast Cancer	Active, not recruiting
**Durvalumab**	Oleclumab + Monalizumab	NCT05061550	II	NSCLC	Recruiting
**Oleclumab + Durvalumab**	–	NCT04262375	II	NSCLC Renal Cell Carcinoma	Withdrawn
IPH520*	Durvalumab + Oleclumab	NCT04261075	I	Advanced Solid Tumors	Completed
AZD4635*	Durvalumab + Oleclumab	NCT04089553	II	Prostate Cancer, mCRPC	Completed
**Chemotherapy Combined with Radiotherapy**	Durvalumab + Oleclumab	NCT03875573	II	Breast Cancer - Luminal B	Active, not recruiting
**Oleclumab + Durvalumab**	–	NCT04262388	II	Pancreatic Ductal Adenocarcinoma, NSCLC, Squamous Cell Carcinoma of Head and Neck	Withdrawn
FOLFOX*	Bevacizumab + Durvalumab + Oleclumab	NCT04068610	Ib/II	Metastatic Microsatellite-stable Colorectal Cancer	Terminated
**Oleclumab**	–	NCT03736473	I	Advanced Solid Malignancies	Completed
**Durvalumab + Oleclumab + Nab-paclitaxel + Gencitabine**	–	NCT04940286	II	Pancreatic Adenocarcinoma	Recruiting
FOLFOX*	Durvalumab + Oleclumab + Monalizumab	NCT04145193	II	Microsatellite-stable Colorectal Cancer	Withdrawn
**Durvalumab**	Oleclumab+ Monalizumab	NCT03822351	II	NSCLC Unresectable	Completed
**Oleclumab**	Durvalumab	NCT02503774	I	Advanced Solid Tumors	Completed
AZD4635*	Durvalumab + Enzalutamide + Oleclumab + Docetaxel + Abiraterone	NCT02740985	I	Advanced Solid Malignancies, NSCLC, mCRPC, Colorectal Carcinoma	Completed
**Durvalumab**	Oleclumab + Monalizumab + Danvatirsen	NCT03794544	II	Resectable NSCLC	Completed
**Durvalumab**	Olaparib + AZD9150 + Vistusertib + Oleclumab + AZD6738	NCT03334617	II	NSCLC	Active, not recruiting
**Oleclumab**	Osimertinib + AZD4635	NCT03381274	I/II	NSCLC	Active, not recruiting
**Durvalumab**	Paclitaxel + Capivasertib + Oleclumab + Trastuzumab deruxtecan+ Datopotamab deruxtecan	NCT03742102	I/II	Triple Negative Breast	Recruiting
**Durvalumab**	Danvatirsen + Oleclumab + MEDI5752 + AZD2936 + Chemotherapy	NCT03819465	I	NSCLC	Active, not recruiting
**Durvalumab**	Monalizumab + Oleclumab + Ceralasertib (AZD6738) + Savolitinib+ Docetaxel	NCT03833440	II	NSCLC	Active, not recruiting
**Oleclumab**	Gencitabine + Durvalumab + FOLFOX + Nab-paclitaxel	NCT03611556	I/II	Metastatic Pancreatic Adenocarcinoma	Completed
**Oleclumab + Durvalumab**	–	NCT04668300	II	Sarcoma	Recruiting
**IBI325**	Sintilimab	NCT05119998	I	Solid Tumor	Completed
**IBI325 +** **Sintilimab**	–	NCT05246995	I	Solid Tumor	Unknown Status
**CPI-006**	Ciforadenant + Pembrolizumab	NCT03454451	I	Solid Tumor + Non-hodgkin Lymphoma	Completed
**Sym024**	–	NCT04672434	I	Solid Tumor + Metastatic Cancer	Active, not recruiting
**PT199**	Anti-PD-1 monoclonal antibody	NCT05431270	I	Solid Tumor	Recruiting
**Dalutrafuspalfa**	mFOLFOX6	NCT04261075	I	Advanced Solid Tumors	Completed
**JAB-BX102**	Pembrolizumab	NCT05174585	I/II	Solid Tumor	Recruiting
**AK119**	AK104	NCT04572152	I	Advanced or Metastatic Solid Tumors	Active, not recruiting
**AK119**	–	NCTT051732	I	Solid Tumor	Active, not recruiting
**TJ004309**	–	NCTT050017	II	Solid Tumor	Active, not recruiting

Non-Small Cell Lung Cancer (NSCLC); Metastatic Castration-Resistant Prostate Cancer (mCRPC). *IPH5201: anti-CD39 blocking monoclonal antibody; AZD4635: small molecule inhibitor of A2AR signaling; FOLFOX: folinic acid, fluorouracil and oxaliplatin; AZD9150: antisense oligonucleotide inhibitor of STAT3; T-DXd: Trastuzumab deruxtecan; Dato-DXd: Datopotamab deruxtecan; AZD6738: Ceralasertib; MEDI5752: PD-1/CTLA-4 bispecific antibody.

## 4 The potential effect of adenosine/CD73 pathway in prostate cancer therapy

The purinergic system, which includes ATP and adenosine signaling pathways, has been shown to play an important role in the development and progression of prostate cancer. Studies have revealed that the activation of purinergic receptors, such as P2R, can enhance the proliferation, migration, and invasion of prostate cancer cells. Studies have shown that ATP can inhibit the growth of prostate cancer cells, likely through P2 receptors (53). As a result, many studies have described the antineoplastic effects of extracellular nucleotides and ectonucleotidases in different types of tumors (54, 55).

CD73 is an independent prognostic factor in prostate cancer, as the expression of CD73 in the prostate epithelium suppresses immunosurveillance by CD8+ T cells, while the expression of CD73 in the tumor stroma reduces NF-kB signaling in tumor cells via adenosine A2B receptor. CD73 expression, even on the normal adjacent prostate epithelium, can thus effectively discriminate between aggressive and indolent forms of prostate cancer (54).

The presence of tumor-infiltrating CD8+ T cells has been associated with a favorable prognosis in various types of cancer (55) and CD73 deficiency is associated with a significant reduction in prostate tumor growth and increased infiltration of CD8+ T cells so CD73 may be associated with prostate cancer progression and decreased antitumor immunity (36). The exosomes are a distinct population of extracellular vesicles, these exosomes CD73+ can directly impair the capacity of CD14+ monocytes to differentiate into functional dendritic cells (DC), thus having a potential effect on both antigen presentation and subsequent T cell responses (56). Consequently, the dominant effect of DC function by prostate cancer exosomes is immunosuppression and not antigen delivery (57).

Currently, there is a focus on understanding the impact of CD39 enzymatic function on immunity, and how therapeutic modulation of this pathway alters its functional potential within the tumor microenvironment (27). Increased levels of CD39 expression have been observed in various solid tumors (23), and extracellular vesicles derived from these cell types also contain CD39 and CD73 are believed to generate adenosine (58). However, further studies are needed to evaluate this association in the context of prostate cancer.

Some studies in patients with metastatic castrate-resistant prostate cancer (mCRPC) showed that the inhibition of the adenosine 2A receptor (A2AR) diminishes the immunosuppressive effects of adenosine and may complement immune-targeting drugs such as durvalumab and oleclumab anti-CD73. However, in this heavily pretreated population, AZD4635 with durvalumab or oleclumab demonstrated minimal antitumor activity. Safety profiles of the treatment combinations were manageable and consistent with the known profiles of the individual agents. They concluded that exploring alternative dosing in less heavily treated patients may be beneficial (59).

Most interestingly, synergistic effects of combination therapy associating CD39 inhibition with immune checkpoint blockade, MAPK inhibitors, or immunogenic chemotherapy were recently reported (60). Furthermore, Gardani et al. (10) reported that the hydrolysis of ATP, ADP, and AMP is increased in the plasma of patients diagnosed with PC. Higher levels of ATP hydrolysis were observed in patients with clinical stage IIA, compared to those with locally more advanced disease, such as in clinical stages IIB and III. In contrast, higher levels of AMP hydrolysis were observed in patients with stages IIB and III, suggesting that the coordinated action of CD39/CD73, phosphodiesterase, and alkaline phosphatase can modulate the immune system, creating a favorable environment for tumor development (10). Besides, another study investigated the expression of CD39 and CD73 enzymes as potential biomarkers in prostate cancer. By analyzing tissue and blood samples from PC patients, the findings suggest that CD39 and CD73 could serve as valuable biomarkers for prostate cancer, aiding in diagnosis and treatment strategies (61).

## 5 Discussion

This review has focused on the role of purinergic signaling in cancer, especially in prostate cancer. We have discussed how modulations of the ectonucleotidases can potentially impact antitumor immunity. Prostate cancer is one of the most common neoplasms and one of the main causes of death in males worldwide. Most patients die due to complications caused by metastases; however, they have long survival periods.

Upon carefully reviewing the scenario of cancer, we recognize the significance of the purinergic system in this process. ATP and adenosine act synchronously, sometimes exhibiting anti-tumor and at other times pro-tumor actions. It is believed that modulating CD39 could have a substantial impact on antitumor immunity by acting in the ATP/ADO cascade and can act in two ways: initially preventing ATP phosphorylation and subsequently preventing the accumulation of ADO in the TME. It is crucial to correlate the actions of ectonucleotidases (CD39/CD73), as the conversion of eATP to AMP by CD39 leads to increased production of ADO, resulting from enhanced stimulation of the CD73 pathway.

However, regarding prostate cancer, we have not yet obtained sufficient data in the literature to conclusively define their behavior. Due to their critical roles as regulators of extracellular adenosine levels, ectonucleotidases are uniquely positioned to regulate cancer growth. Within this complex landscape, the adenosine/CD73 pathway plays a crucial role. Additionally, CD73 has been demonstrated to be an independent prognostic factor in prostate cancer, and several clinical trials using anti-CD73 immunotherapy are currently underway. There is also growing interest in the potential pharmacological manipulation of the adenosine/CD73 pathway in prostate cancer patients.
